# MCP‐1 From Aging Dermal Adipose Tissue Impairs Hair Follicle Cycling via Macrophage Recruitment: Implications for Age‐Related Alopecia

**DOI:** 10.1111/acel.70799

**Published:** 2026-11-08

**Authors:** Jian Chen, Yuhui Hou, Ke Ye, Yilong Guo, Pingping Sun, Fangfang Qi, Zhexiang Fan, Damao Dai, Xiaomin Liu, Shulian Wu, Demengjie Le, Zhiqi Hu, Yong Miao, Qian Qu

**Affiliations:** ^1^ Department of Plastic and Aesthetic Surgery Nanfang Hospital of Southern Medical University Guangzhou China; ^2^ Department of Plastic and Aesthetic Surgery, Zhujiang Hospital Southern Medical University Guangzhou China; ^3^ Department of Plastic and Cosmetic Surgery, Shenzhen People's Hospital, Second Clinical Medical College of Jinan University Medical College 1st Affiliated Hospital of Southern University of Science and Technology Shenzhen China; ^4^ Department of Plastic and Aesthetic Surgery Clifford‐Hospital Guangzhou China

**Keywords:** aging, dermal white adipose tissue, hair regeneration, macrophages, melanogenesis

## Abstract

Aging leads to significant hair changes, including alopecia and graying, yet the underlying mechanisms remain unclear. Emerging evidence highlights the role of the extracellular microenvironment, particularly dermal white adipose tissue (dWAT), in regulating hair regeneration and pigmentation. Previous studies showed that young murine dWAT improves age‐related hair phenotypes via follistatin upregulation. This study investigates key molecular mechanisms in dWAT governing hair aging, aiming to inform targeted therapies for age‐related hair disorders. Using in vivo murine models, protein arrays, FACS, immunofluorescence, and transcriptomics, we identified monocyte chemoattractant protein‐1 (MCP‐1) as a critical factor secreted by aged dWAT. MCP‐1 recruits macrophages to perifollicular regions. Depleting skin macrophages in aged mice shifted hair follicles from telogen to anagen and upregulated melanocyte‐lineage proteins, including DCT, Tyrosinase‐related protein 1 (TYRP1), and Pmel17. Transcriptomic analysis revealed macrophage absence activated signaling pathways essential for hair growth and melanogenesis, upregulating hair follicle development genes. Conversely, M0 and M2 macrophages were associated with earlier anagen entry, whereas M1 macrophages delayed anagen transition. These findings identify dWAT as a pivotal regulator of age‐associated hair changes, in which MCP‐1–mediated macrophage accumulation is critically involved.

## Graphical abstract

Aging orchestrates a dWAT–MCP‐1–CCR2 axis that recruits and reprograms macrophage subpopulations, disrupting the local M1/M2 balance and suppressing WNT signaling, thereby impairing both hair cycle transition and production of genes associated with melanogenesis.

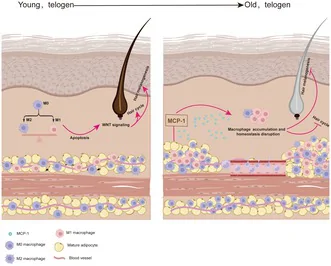

## 1 Introduction

Dermal white adipose tissue (dWAT) is a special type of adipose tissue that surrounds hair follicle (HF) and is a key modulator of the skin microenvironment. dWAT functions when skin responds to bacterial infections and during the process of wound healing (Zwick et al. 2018). Studies of various mouse models with defects in dWAT revealed that this tissue is indispensable for hair growth (Stone et al. 2004; Eric et al. 2011; Sardella et al. 2018). Different adipocytes in dWAT secrete regulatory factors, forming a complex external network with the dermis and epidermis (Plikus et al. 2008) to influence hair cycle, regeneration, and pigmentation (Zwick et al. 2018; Nicu et al. 2021). In addition, dWAT has notable functions in aging‐associated alopecia. Aging dWAT overexpresses inhibitory signals BMP2 (Bone Morphogenetic Protein 2), DKK1 (Dickkopf‐related protein 1), and SFRP4 (Secreted Frizzled‐Related Protein 4) during early anagen and expresses a reduced level of a facilitative signal (follistatin) during late telogen/early anagen, which represses hair cycling in aging mice (Eric et al. 2011; Chen et al. 2014). Nishimura et al. recently reported that obesity‐induced stress targets HF stem cells (HFSCs) for epidermal keratinization and thereby accelerates hair thinning (Morinaga et al. 2021), which is similar to the process of characteristic changes in hair during aging. The mechanism underlying the close association between dWAT and HF changes during aging remains unclear. Adipose‐derived therapies are an important emerging strategy for hair regeneration (Klinger et al. 2008; Mojallal et al. 2009; Cho et al. 2010; Brongo et al. 2012; Dini et al. 2014; Akdag et al. 2018; Wang, Dai, et al. 2019; Wang, Liao, et al. 2019). In‐depth exploration of the regulatory mechanism linking dWAT and aging‐associated hair loss and white hair can not only improve understanding of HF aging but also provide new treatment ideas.

Previous work has suggested that aging dWAT is accompanied by increased expression of inflammatory factors such as CXCL1 (C‐X‐C motif chemokine ligand 1), monocyte chemoattractant protein‐1 (MCP‐1), and TNFα (Tumor Necrosis Factor‐Alpha), which results in a chronic inflammatory microenvironment around HFs (Akdag et al. 2018; Chen, Wu, et al. 2021). Exposure to high levels of pro‐inflammatory cytokines such as TNFα and IL‐1β (Interleukin‐1 Beta) induces apoptosis of hair bulb keratinocytes in vivo (Ruckert et al. 2000). Moreover, obesity promotes hair thinning through IL‐1R (Interleukin‐1 Receptor) signaling (Morinaga et al. 2021). Therefore, we speculated that inflammation in dWAT is one of the most important factors leading to hair changes during aging. Here, we found that pioglitazone treatment reduced several inflammatory mediators in aged dWAT and was accompanied by earlier anagen entry. Therefore, we performed a series of experiments to investigate whether inflammatory mediators in aged dWAT are associated with impaired hair follicle regeneration. Among the altered factors, MCP‐1 was identified as a key upregulated molecule. MCP‐1 neutralization in aged skin was associated with enhanced anagen entry and reduced macrophage accumulation. Macrophage accumulation correlated with attenuated hair cycle progression, decreased melanogenesis, and downregulation of Hedgehog, Wnt, and cell cycle–related pathways.

## 2 Results

### 2.1 Pioglitazone Treatment Is Associated With Reduced MCP‐1 and Earlier Anagen Entry

Pioglitazone is a PPARγ (Peroxisome proliferator‐activated receptor) agonist that upregulates adiponectin, promotes adipocyte differentiation, and alleviates adipose tissue inflammation (Shimano et al. 2008; Sato et al. 2011; Chen, Fan, et al. 2021; Chen, Wu, et al. 2021). After 30 days of daily injections, pioglitazone promoted anagen entry in the dorsal skin of aged mice, which occurred earlier than in vehicle‐treated controls (Figure 1A–D). To assess the effect of pioglitazone on inflammatory mediators in dWAT, we performed a Luminex‐based multiplex assay (Bio‐Plex Pro Mouse Cytokine/Chemokine 23‐plex panel) to quantify cytokine and chemokine levels in dWAT from pioglitazone‐treated and control mice. Notably, pioglitazone treatment in aged mice was associated with significant reductions in MCP‐1, GM‐CSF (granulocyte‐macrophage colony‐stimulating factor), and TNFα, with no comparable changes detected in young mice. Among these factors, MCP‐1 exhibited the most significant reduction in pioglitazone‐treated old mice (Figure 1E,F, Figure S1). Immunofluorescence staining revealed increased expression of the hair growth marker β‐catenin and the proliferation marker Ki67 in the hair bulbs of aged mice treated with pioglitazone (Figure 1G–I). Collectively, these findings indicate that pioglitazone treatment is associated with reduced MCP‐1 and earlier anagen entry. Pioglitazone induces adipogenesis in dWAT, a process previously linked to attenuated inflammation and changes in follicle morphology. To determine whether pioglitazone's effect on hair cycling depends on this adipogenic action, mice were co‐treated with a PPARγ antagonist. Mice were divided into four groups: control, pioglitazone alone, PPARγ antagonist alone, and pioglitazone plus antagonist. Compared with the control and antagonist‐only groups, both the pioglitazone group and the combination group entered anagen earlier (Figure S2A,B). No significant difference in dWAT thickness was observed between aged pioglitazone‐treated and control mice, whereas dWAT was significantly thinner in both the PPARγ antagonist alone and pioglitazone‐plus‐antagonist groups compared with controls (Figure S2C,D). Consistently, MCP‐1 immunostaining showed reduced MCP‐1 levels in the pioglitazone group and the combination group relative to the others, with no significant difference between them (Figure S2E,F). Serum adiponectin levels were measured after intraperitoneal (i.p.) and intradermal (i.d.) pioglitazone administration. Systemic i.p. injection significantly elevated adiponectin compared with controls, whereas local i.d. injection did not substantially alter circulating adiponectin levels (Figure S2G). Notably, i.d. treated mice showed comparable serum adiponectin levels between the pioglitazone‐only and pioglitazone‐plus‐antagonist groups. Despite the absence of a marked adiponectin increase in either i.d. group, both routes of administration triggered precocious anagen entry, as revealed by gross examination. As adiponectin is a key mediator of adipogenesis, these results raise the possibility that pioglitazone promotes hair follicle cycling through mechanisms extending beyond its pro‐adipogenic activity. Given the prominent MCP‐1 changes following pioglitazone treatment, we further speculate whether MCP‐1 in dWAT might be sufficient to induce the characteristic hair changes observed during aging—a hypothesis that remains to be directly tested.

**FIGURE 1:**
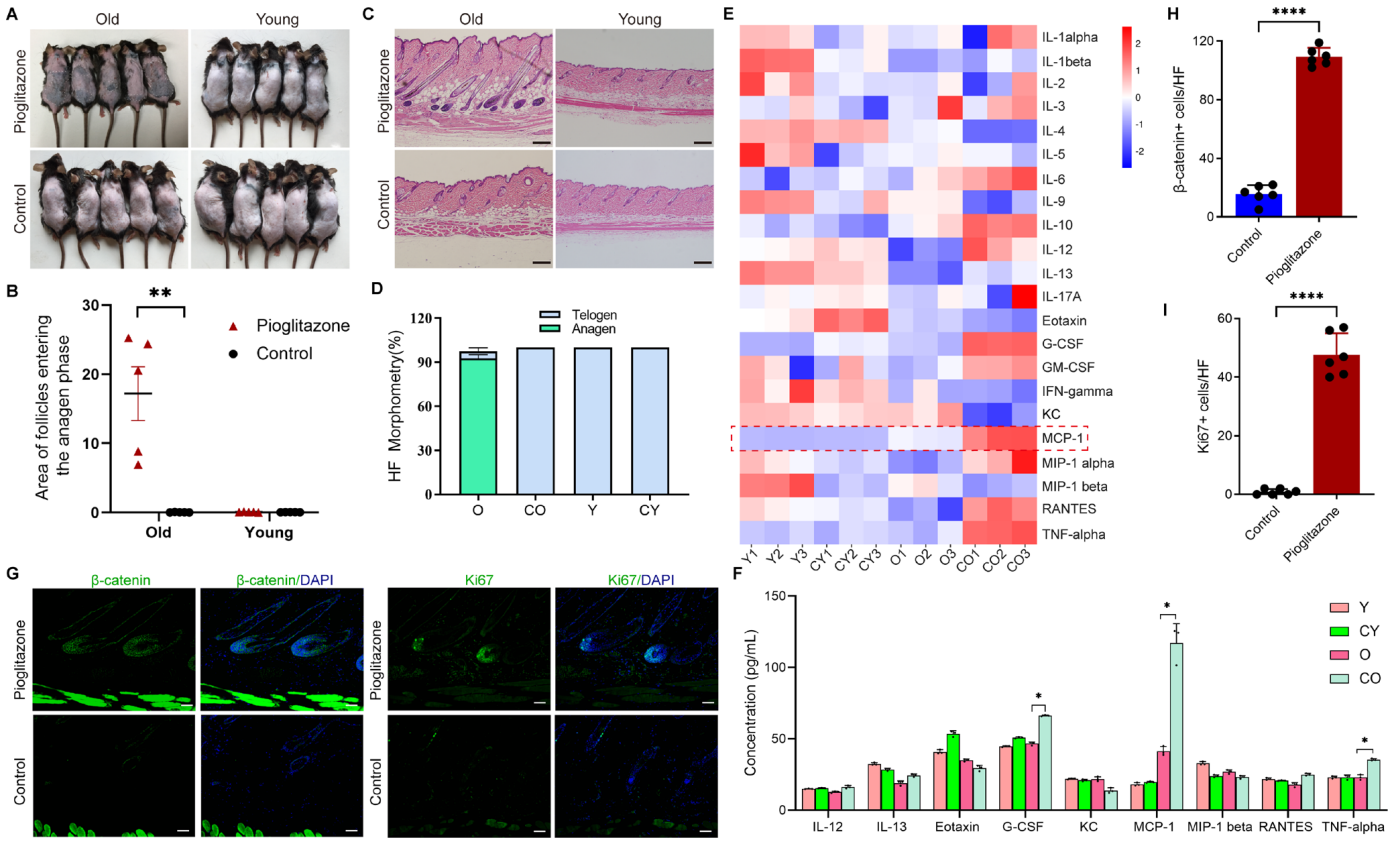
Pioglitazone treatment is associated with reduced MCP‐1 and earlier anagen entry. (A) Representative images of mice administered pioglitazone for 30 days. *n* = 5 mice per group. (B) Area of hair follicles entering anagen after pioglitazone or vehicle treatment. *n* = 5 mice per group. (C, D) Representative H&E‐stained dorsal skin sections and quantitative morphological analysis of hair follicles, (*n* = 3 mice per group). Images show old control, young control, old pioglitazone‐treated, and young pioglitazone‐treated mice at Day 30 post‐treatment. Scale bars = 200 μm. (E, F) Results of a mouse cytokine/chemokine 23‐plex array panel in skin of young and old mice administered pioglitazone or vehicle for 1 month. *N* = 3 mice per group, (G) Immunofluorescence analysis of the hair growth markers β‐catenin and Ki67 in the old pioglitazone‐treated and old control group at Day 30. Scale bars = 100 μm. *N* = 8 (from 3 mice per group). (H, I) Quantification of cells positive for β‐catenin and Ki67, *n* = 6 (from 3 mice per group). Scale bars = 100 μm. unpaired two‐tailed Student's *t*‐test. *p < 0.05, **p < 0.01, ****p < 0.0001. ns, not significant. CO, the dWAT of old mice after injection with vehicle; CY, the dWAT of young mice after injection with vehicle; O, the dWAT of old mice after injection with pioglitazone; Y, the dWAT of young mice after injection with pioglitazone.

### 2.2 Local MCP‐1 Neutralization During Telogen Promotes Anagen Entry in Aged Mice

MCP‐1 exhibited significant upregulation during telogen and emerged as the most prominently downregulated cytokine in dWAT of aged mice treated with pioglitazone. Our focus on MCP‐1 aimed to unravel its connection with age‐related hair changes. Initially, we intradermally administered the neutralizing antibody targeting MCP‐1 (Anti‐MCP‐1), effectively triggering local telogen‐to‐anagen transition in aged mice (Figure 2A,B). Notably, the abundance of F4/80^+^ macrophages markedly decreased (Figure 2C,D). However, the neutralization of MCP‐1 did not significantly affect the hair follicle cycle or macrophage numbers in the dorsal skin of young mice (Figure 2A,B,G,H). MCP‐1 is a well‐established mediator of monocyte recruitment and macrophage accumulation in adipose tissue (Kamei et al. 2006; Kanda et al. 2006). We therefore tested whether macrophages serve as the cellular effectors through which MCP‐1 influences hair follicle structure and regeneration. Our results demonstrated that MCP‐1‐injected mice experienced a delayed anagen entry compared to control mice, indicating MCP‐1's negative regulation of hair growth (Figure 3A–C). Moreover, using clodronate liposomes to induce macrophage apoptosis (M‐Clo group) after MCP‐1 injection (MCP‐1 + M‐Clo group) successfully reversed the inhibitory effects of MCP‐1 (Figure 3A–F): The number and total area of hair follicles entering anagen in the dorsal skin were significantly reduced in the MCP‐1 group compared with both the MCP‐1 + M‐Clo and control groups. In contrast, no significant differences in either the number or total area of anagen follicles were observed between the MCP‐1 + M‐Clo group and the control group (Figure 3A–D). The MCP‐1 treatment group exhibited a significantly reduced hair bulb diameter and shortened hair follicle length compared with both the control group and the MCP‐1 + M‐Clo group (Figure 3E,F). Immunofluorescence analyses revealed that MCP‐1 administration reduced β‐catenin and Ki67 expression, with no significant differences in Ki67^+^ and β‐catenin^+^ cell levels between vehicle‐treated and MCP‐1 + M‐Clo groups (Figure 3G–I). Intradermal MCP‐1 injection significantly elevated F4/80^+^ macrophage levels, particularly in dWAT (Figure 2I,J). These findings suggest that macrophages may function as downstream effectors of MCP‐1, contributing to the prevention of hair growth.

**FIGURE 2:**
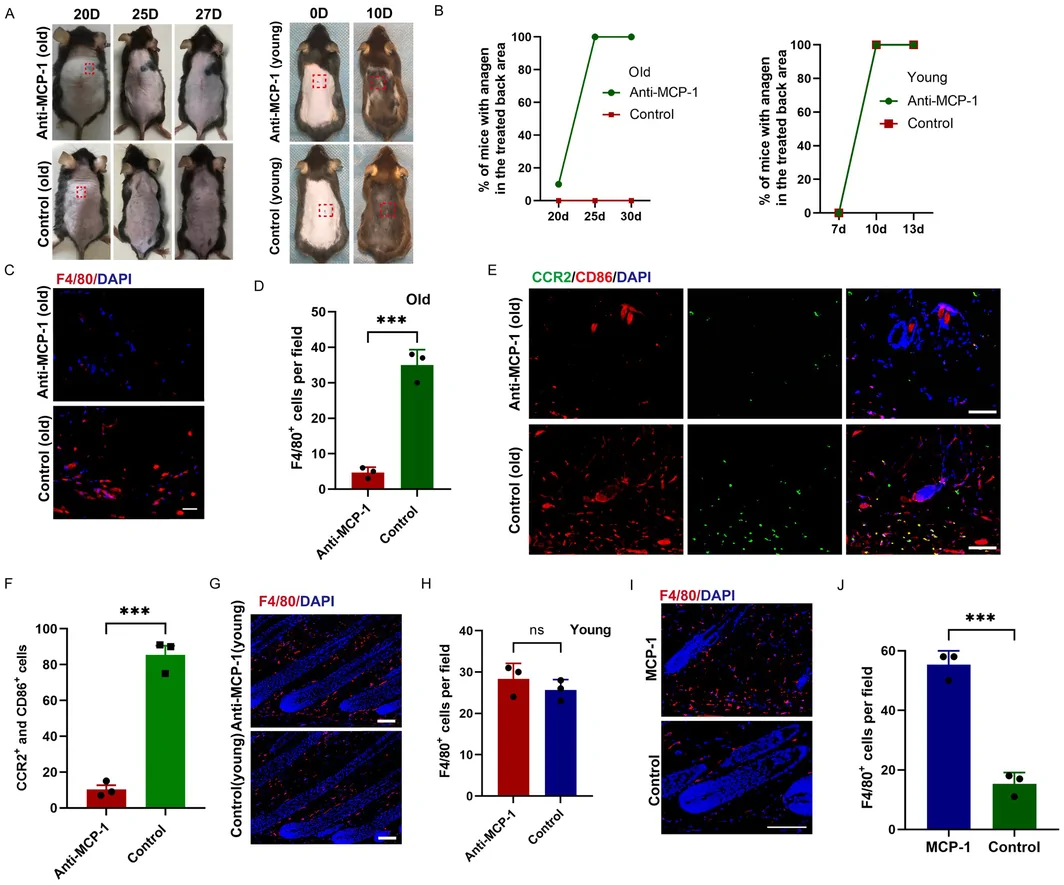
Anti‐MCP‐1 treatment was associated with rapid anagen induction in the treated back area of old mice and reduction the number of skin macrophage. (A) Representative dorsal images of aged and young mice treated with anti‐MCP‐1 or vehicle at the indicated time points. Red dashed line outlines the treatment area; black marks indicate injection sites. (B) Percentage of aged and young mice showing anagen entry in the treated back area after anti‐MCP‐1 or vehicle treatment (*n* = 3 per group). (C, D, G, H) Representative immunofluorescence images and quantitative analysis of F4/80^+^ macrophages in the skin of aged and young mice treated with anti‐MCP‐1 or vehicle. *n* = 3, Scale bars, 20 μm. (E, F) Co‐localization and quantification of CCR2^+^ and CD86^+^ cells in the skin of aged mice treated with anti‐MCP‐1 or vehicle. *n* = 3, Scale bars = 50 μm. (I, J) Representative immunofluorescence images and quantification of F4/80^+^ macrophages in young mouse skin after MCP‐1 or vehicle treatment. Scale bars, 50 μm. *n* = 3 mice per group. ***p < 0.001; ns, not significant.

**FIGURE 3:**
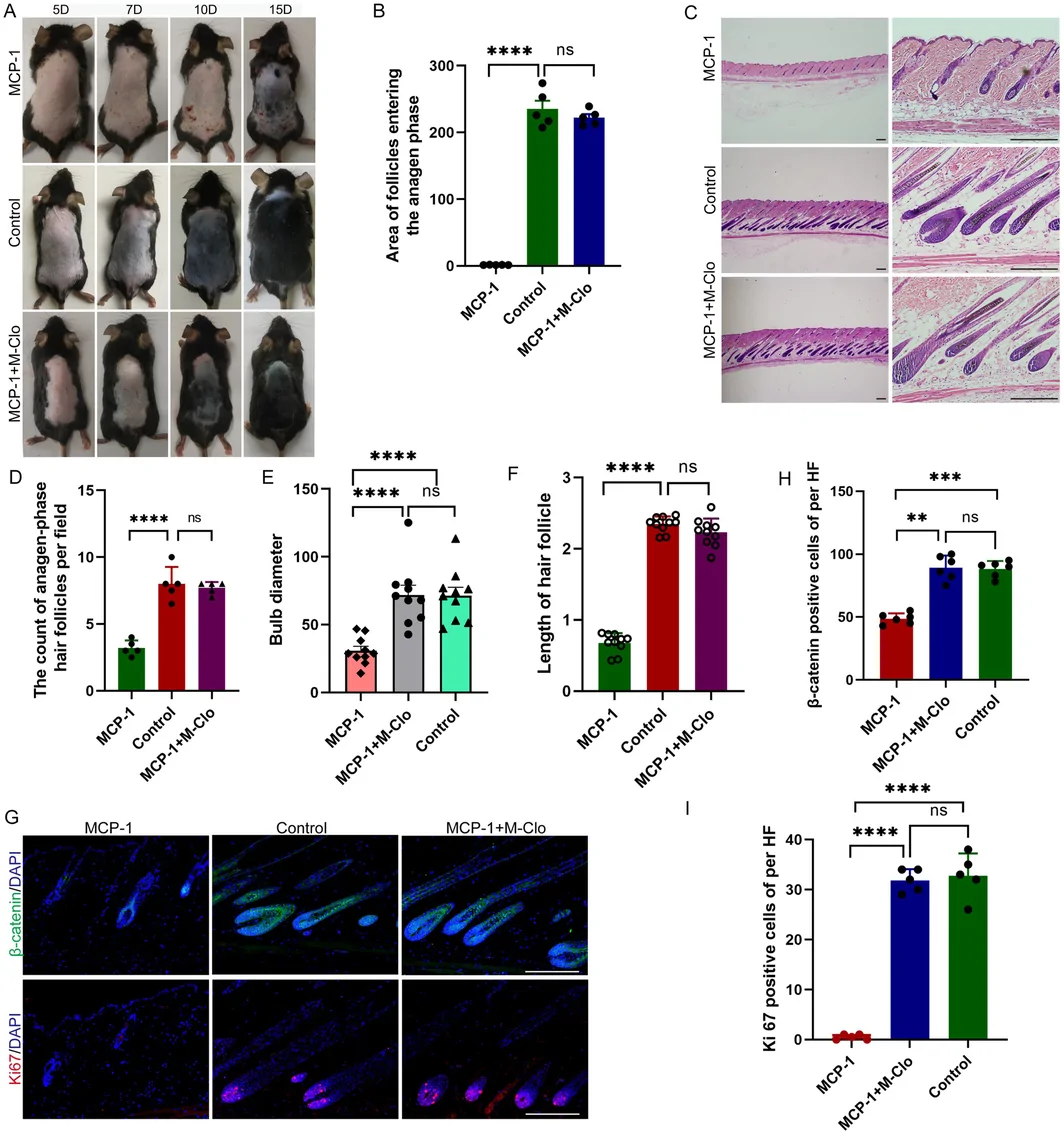
MCP‐1 inhibits hair regrowth and promotes macrophage recruitment to the dWAT. (A) Representative dorsal skin images of young mice treated with MCP‐1, vehicle, or MCP‐1 combined with clodronate liposomes at days 5, 7, 10, and 15. (B) Percentage of the treated back area entering anagen on Day 10 after MCP‐1, MCP‐1 plus clodronate liposomes, or vehicle treatment. n=5micepergroup. (C) H&E‐stained sections at Day 15 in mice treated with MCP‐1, MCP‐1 plus clodronate liposomes, or vehicle. Scale bars, 100 μm. (D–F) Follicle length (*n* = 10), bulb diameter (*n* = 9), and anagen follicle count (*n* = 5 per group) were quantified in each group. (G) Representative immunofluorescence staining for Ki67 and β‐catenin in the skin on Day 15. Scale bars, 200 μm. (H, I) Quantification of Ki67^+^ (*n* = 5 biological replicates per group) and β‐catenin^+^ cells (*n* = 6 biological replicates per group) per hair follicle. Scale bars, 100 μm. ***p < 0.001; ****p < 0.0001; ns, not significant.

### 2.3 Macrophages Are Recruited by MCP‐1 and Accumulate in dWAT During Aging

Immunofluorescence analysis of F4/80 in old and young mouse skin showed that macrophage counts significantly increased in old mouse skin during the telogen phase compared to young mice (Figure 4A,B). To identify which macrophage populations were altered, single‐cell suspensions were prepared from whole skin—with the subcutaneous white adipose tissue (sWAT) removed and the dermal white adipose tissue (dWAT) retained—and analyzed by fluorescence‐activated cell sorting (FACS). The total macrophage count increased with aging, constituting 7.9% and 1.1% in old and young mice, respectively. Furthermore, quantitative analysis showed that aging led to a significant enrichment of M1‐like macrophages (88.3% ± 7.51% in old vs. 34.1% ± 2.84% in young mice, *p* < 0.01), whereas the percentage of M2 macrophages remained statistically unchanged (12.6% vs. 5.3%, *p* > 0.05) (Figure 4C,D). Despite numerical increases in both subsets, the surge in M1 macrophages was the predominant and statistically robust feature of the aged microenvironment (Figure 4D). We reasoned that aging drives macrophage recruitment through elevated MCP‐1 signaling. Supporting this, we found that CCR2, the cognate receptor for MCP‐1, was upregulated in aged skin and largely co‐localized with F4/80^+^ macrophages in both dWAT and around aged hair follicles (Figure 4E,F). To further test whether MCP‐1 signaling promotes the accumulation of M1‐like macrophages, we performed immunofluorescence co‐localization staining for CD86, a marker of M1‐like macrophages, and for CCR2 on skin sections from aged mice treated with an MCP‐1 neutralizing antibody and from control mice. In control aged skin, CCR2 co‐localized with CD86‐positive cells, whereas the number of CCR2/CD86 double‐positive cells was markedly reduced in the skin of the anti‐MCP‐1 group compared with controls (Figure 2E,F). These findings suggest that lowering MCP‐1 levels in the skin reduces the abundance of M1‐like macrophages. Therefore, MCP‐1 in aged dWAT disrupts macrophage homeostasis. Multiple studies have established that the CCR2/CCL2 axis governs the recruitment of pro‐inflammatory monocytes to adipose tissue in obesity (Weisberg et al. 2006; Kanda et al. 2006; Li et al. 2008), and this pathway likely operates similarly in aging. Monocyte transportation from blood to inflamed tissue relies on CCR2 and MCP‐3 (Monocyte Chemoattractant Protein‐3), with CCR2 playing a pivotal role in obesity‐associated adipose tissue inflammation and macrophage accumulation (Weisberg et al. 2006; Li et al. 2008; Leslie et al. 2015), illustrating that macrophages in aged skin accumulate in an MCP‐1/CCR2–dependent manner and may originate from blood monocytes.

**FIGURE 4:**
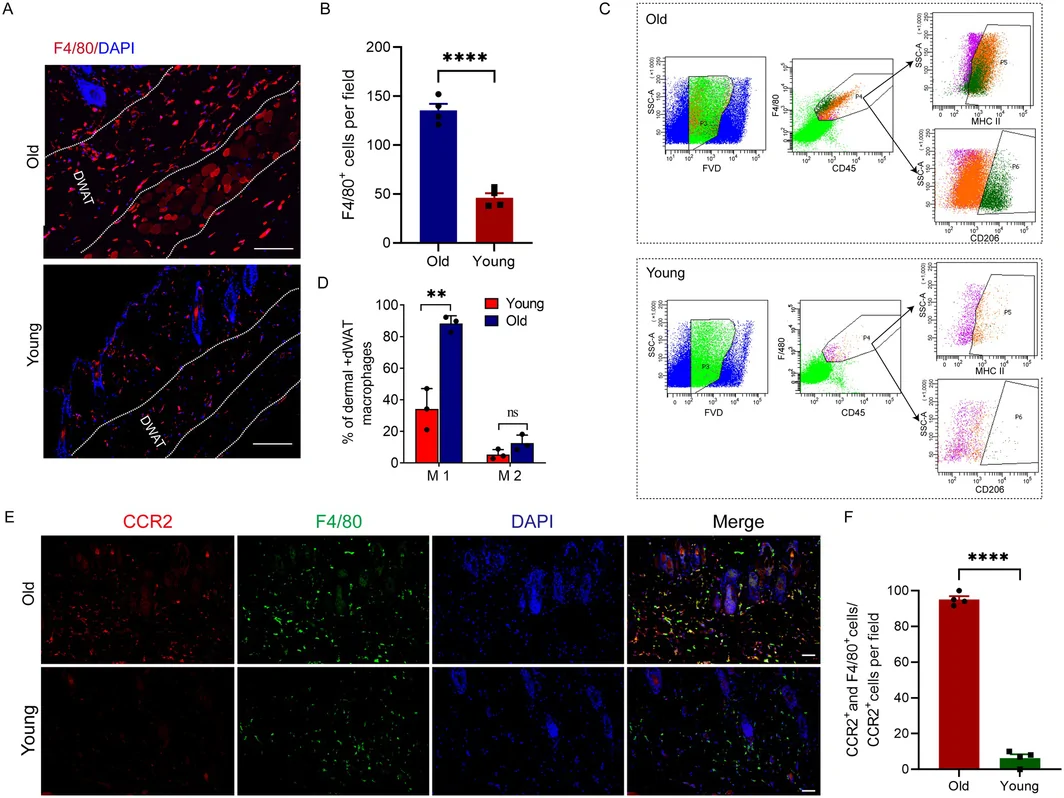
CCR2^+^ macrophages increased in the old mice skin. (A) Immunofluorescence images of F4/80^+^ macrophages in old and young mouse skin. Scale bars = 200 μm. (B) Quantification of F4/80^+^ macrophages in the old and young mice dorsal skin, *n* = 3 field per group; (C) Flow cytometric analysis of old and young mouse skin in telogen phase to isolate M1 (F4/80^+^ CD45^+^ MHCII^+^ CD206^−^) and M2 (F4/80^+^ CD45^+^ MHCII^−^ CD206^+^) macrophages from whole skin that removed SWAT. (D) Quantification of M1 and M2 macrophages in the skin of old and young mice by flow cytometric analysis, *n* = 3 per group. (E, F) Co‐localization and quantification of CCR2^+^ and F4/80^+^ cells in old and young mouse skin. Scale bars = 50 μm. **p < 0.01; ****p < 0.0001; ns, not significant. DWAT, dermal white adipose tissue; SWAT, subcutaneous white adipose tissue.

### 2.4 Macrophages Recruited by MCP‐1 Promote Aging Associated Hair Loss and Melanogenesis

To further explore the functional role of macrophages in hair regeneration during aging, we administered clodronate liposomes to deplete macrophages from skin of old mice (M‐Clo group). After injection, F4/80^+^ cells were significantly reduced in dWAT (Figure 5A,B) and HFs entered anagen at an average of Day 11, aligning with Castellana et al.'s findings, while the hair follicles in the control group remained in the telogen phase until Day 15 (Figure 5C,D). These findings suggest that macrophage accumulation in aged skin contributes to delayed anagen entry. Moreover, regenerated hairs in clodronate liposome‐treated mice showed higher hair counts and a higher pigmentation index than those of control mice (Figure 5E–G). In addition, the level of P16, an important aging‐associated protein, was also reduced in clodronate liposome–treated mice (Figure 5H,I). Further, RNA‐Seq of dermal tissue in treated and control mice aimed to uncover specific mechanisms influencing hair regeneration and cycle transition. A variety of genes associated with the cell cycle were upregulated upon clearance of macrophages (Figure S3). Hair keratin genes such as Krt73, Krt84, Krt27, Krt82, and Krt31, which are related to the hair shaft and inner root sheath formation, were prominently upregulated in clodronate liposome‐treated old mice (Figure 6A, Figure S4). Furthermore, the Dlx3 gene, expressed in HF transit‐amplifying cells, the inner root sheath, and the hair shaft, saw significant upregulation (Figure S4; Zhang and Hsu 2017). Clodronate liposome treatment also upregulated genes related to the melanogenesis pathway, including Mc1r, and prominently increased melanogenesis‐related genes (Tyr, Dct, Tyrp1, Kit, Pmel, Sox5, and Tcf7) in old mice's skin (Figure S4).

**FIGURE 5:**
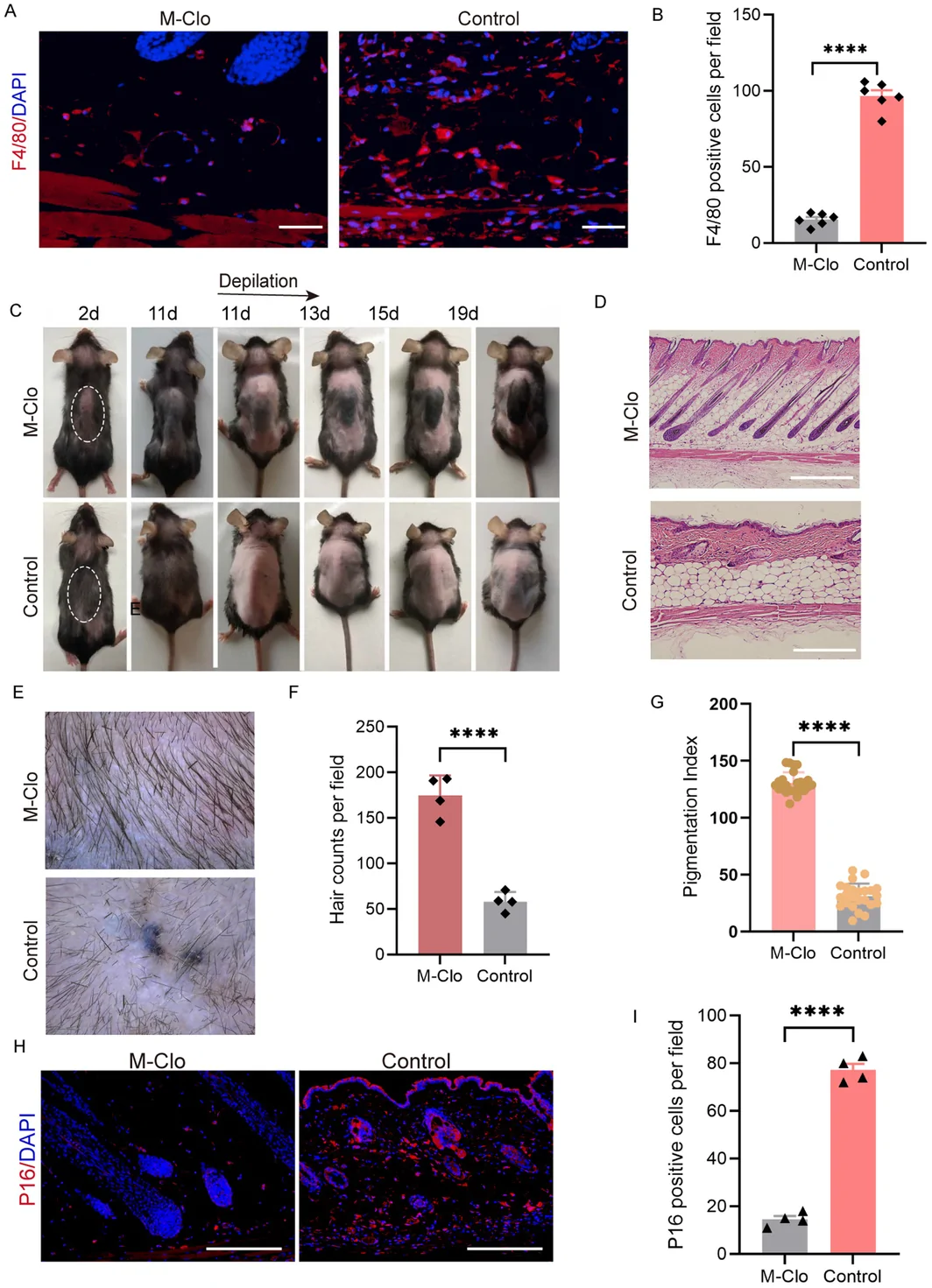
Depletion of macrophages from aging skin induces hair follicle regeneration. (A) Immunofluorescence images of F4/80^+^ macrophages in the dWAT after subcutaneous injection with clodronate liposomes (M‐Clo) and control liposomes on the Day 13. Scale bar, 40 μm. (B) Quantitative analysis of the F4/80^+^ macrophages in the two groups, *n* = 6 fields per group. (C) Representative images of old mice at different days after treatment with M‐Clo and control liposomes, and the mice were depilated on the Day 11 after treatment for convenient observation. The white dotted area is the injection area. (D) Histological H&E staining of the two groups on Day 13 after injection. Scale bar, 500 μm. (E) Trichoscopy examination of the two groups on Day 60. (F, G) Quantification of hair count (4 fields per group) and pigmentation index (24 hair shafts from 6 mice per group) on Day 60. (H, I) Immunofluorescence images and quantitative analysis (*n* = 4) of P16 in the skin of the two groups on the Day 13. Scale bar, 200 μm. ****p < 0.0001.

**FIGURE 6:**
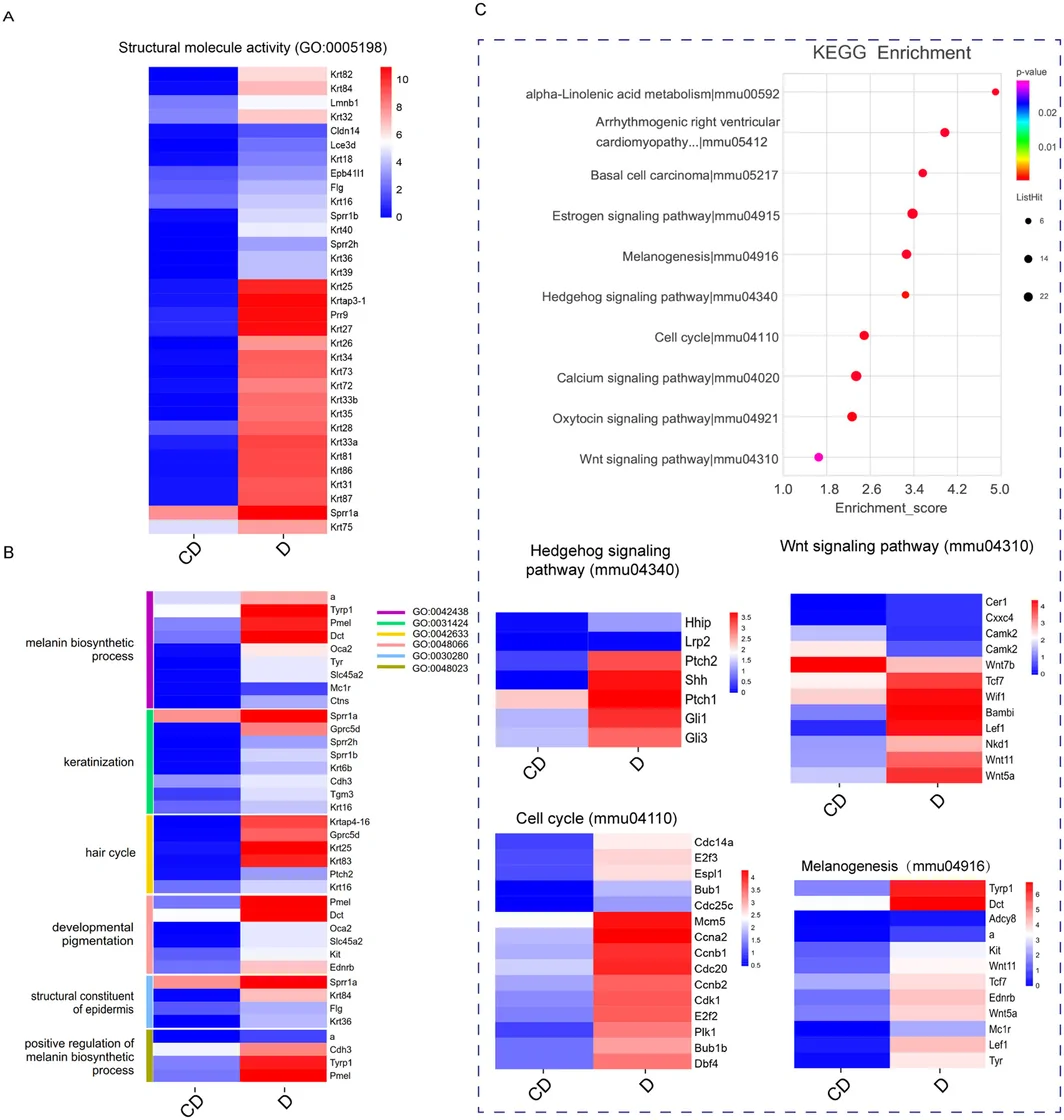
(A–C) RNA‐Seq analysis of the dermis following depletion of macrophages in aging mice and control group, *n* = 3biological replicates. (A, B) Heatmap of significantly enriched GO terms in the dermis of old mice treated with clodronate liposomes compared with control liposomes (*n* = 3 mice per group, independent biological samples). (C) Bubble chart of KEGG enrichment pathways that significantly differed between the dermis of old mice treated with clodronate liposomes and the dermis of old mice treated with control liposomes, and the heat maps of relative differences in expression of genes related to the Hh, cell cycle, Wnt, and melanogenesis signaling pathways between the two groups. CD, dermis of old mice treated with control liposomes; D, dermis of old mice treated with clodronate liposomes. Differentially expressed genes (DEGs) were identified with DESeq (estimate Size Factors and nbinomTest), using thresholds of FDR < 0.05 and a fold change > 2 or < 0.5.

Top Gene Ontology (GO) terms associated with the upregulated genes revealed connections to the cell cycle, cell division, mitotic cell cycle, keratinization, melanin biosynthetic processes, developmental pigmentation, and the hair cycle (Figure S4, Figure 6B). Wnt and melanogenesis signaling pathways, crucial for hair stem cell activation and pigmentation, were highly correlated and upregulated post‐macrophage depletion, as indicated by Kyoto Encyclopedia of Genes and Genomes (KEGG) pathway analysis (Figure 6C). There were 12 upregulated genes, including Wnt5a, Wnt11, LEF1, Wif1, and Bambi, involved in the Wnt pathway, and 12 upregulated genes involved in the melanogenesis pathway. Rittie et al. (2009) demonstrated the involvement of the Hh signaling pathway in maintaining the HFSC phenotype throughout life, and our findings showed its activation upon macrophage elimination from old skin. Immunofluorescence analyses revealed that cells positive for the HFSC activator β‐catenin and Ki67 were extensively found in hair bulbs of old mice upon depletion of macrophages (Figure 7A–C). Depletion of skin macrophages significantly upregulated the expression of melanocyte marker genes and melanogenesis‐related proteins, including DCT, TYRP1, and Pmel17, compared with control skin (Figure 7D–I). To exclude the influence of natural hair cycle variation, we compared macrophage‐depleted anagen skin with untreated aged mice in the same cycle stage. Immunofluorescence staining for TYRP1 and Pmel17 revealed that macrophage depletion markedly increased the number of positive cells compared with control anagen follicles (Figure 7E,F,H,I). Macrophage depletion is associated with increased expression of melanogenesis‐related markers and proteins, which may contribute to enhanced melanin synthesis, providing a potential avenue for promoting hair repigmentation.

**FIGURE 7:**
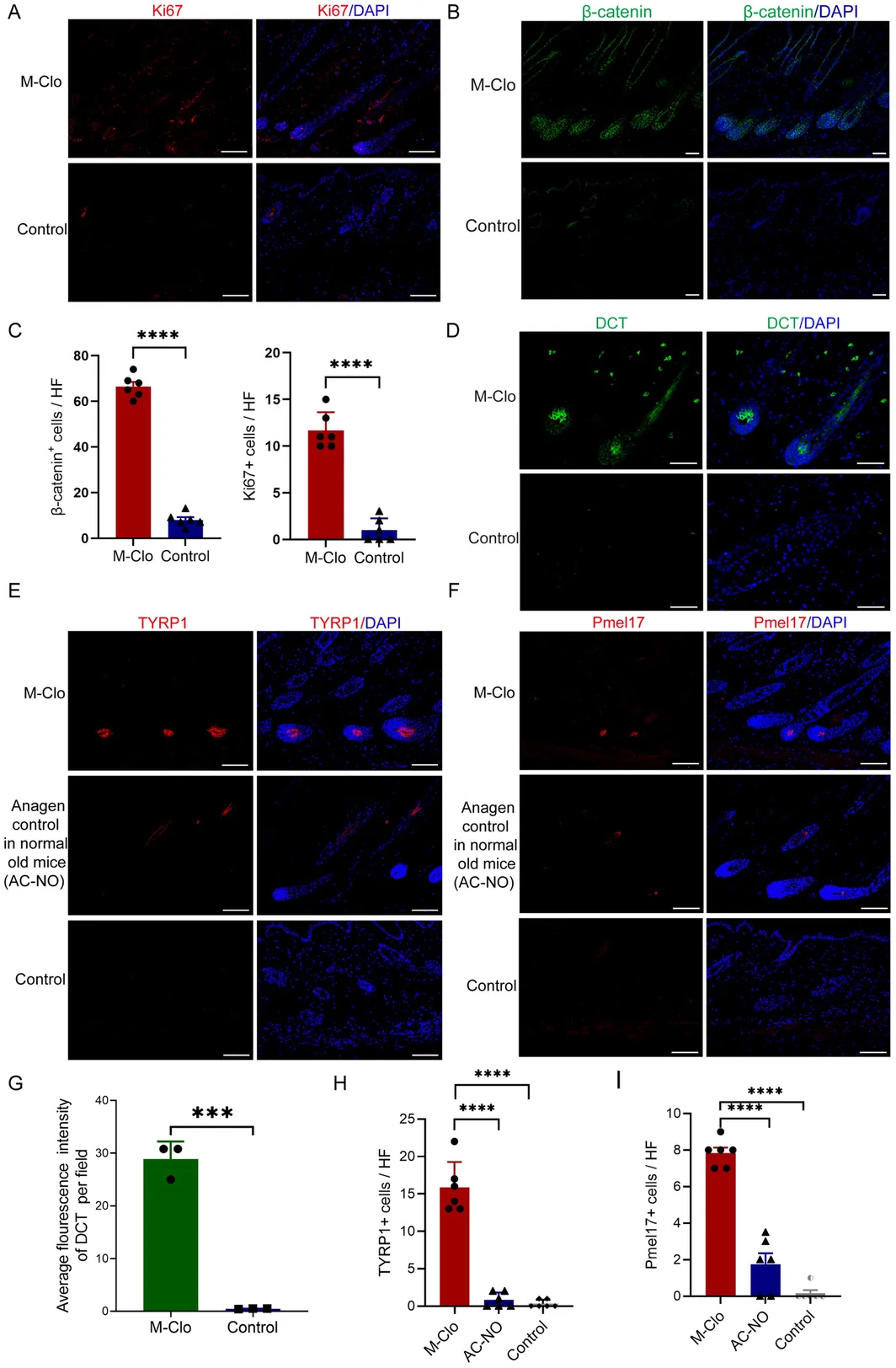
Depletion of macrophages from aging skin induces hair follicle regeneration and melanogenesis‐related proteins. (A, B) Immunofluorescence images of Ki67 (scale bar, 100 μm) and β‐catenin (scale bar, 50 μm) in the skin of old mice treated with M‐Clo and control liposomes at Day 13. (C) Quantification of cells positive for β‐catenin and Ki67 in HFs in the M‐Clo and control liposomes groups, *n* = 6 biological replicates. (D) Immunofluorescence images of DCT in skin of the M‐Clo and control liposomes at Day 13. Scale bar, 50 μm. (E, F) Immunofluorescence staining of TYRP1 and Pmel17 in M‐Clo group, control group, and AC‐NO (anagen control in normal‐old mice) group (due to the fact that the control group was still in telogen phase at the time of sampling, in order to objectively compare the expression of melanin related genes in HFs of the same hair growth cycle, immunofluorescence staining of the elderly mice skin in anagen phase were also performed and analyzed). (G–I) Quantification of cells positive for DCT, TYRP1 and Pmel17 in the different groups, *n* = 3–6 biological replicates. ***p < 0.001 and ****p < 0.0001. AC‐NO, anagen control in normal old mice.

### 2.5 Accumulated M1 Macrophages Inhibit Hair Regeneration and Melanogenesis

To further identify which type of macrophages inhibit HF regeneration and pigmentation, we harvested bone marrow‐derived macrophages (BMDMs) and polarized them into M1 or M2 subtypes using IL‐4, LPS, and IFN‐γ. These cells were then transplanted into old mice (22 months) during the telogen phase. M2 and unpolarized macrophages induced hair regeneration, and HFs entered anagen slightly earlier upon transplantation of unpolarized macrophages compared to M2 macrophages. In contrast, no hair regrowth was observed in mice receiving M1 macrophages. Similarly, the non‐grafted dorsal areas of M0‐ and M2‐transplanted mice, as well as PBS‐injected controls, remained in telogen even at Day 60 post‐treatment (Figure 8A,B). Quantitative hair cycle histomorphometry indicated that more HFs entered anagen following transplantation of M2 macrophages than unpolarized macrophages (Figure 8C). Furthermore, M0 macrophages induced rapid catagen/telogen transition and significant hair loss, whereas the M2 group maintained a prolonged anagen phase with minimal hair loss (Figure 8A–C). Given that 22‐month‐old mice are in an advanced aged stage characterized by a markedly prolonged telogen and impaired regenerative capacity, the control and non‐treated areas failed to enter anagen even by Day 60 post‐transplantation (Figure 8A–C). Therefore, 18‐month‐old mice were introduced as a younger, additional model. Notably, both the control and non‐grafted regions showed a clear tendency to initiate anagen by Day 13 after transplantation, whereas the M1 macrophage–grafted areas remained arrested in telogen (Figure S5). These observations suggest that transplanted M1 macrophages suppress morphological transition of hair follicles from telogen to anagen. To further investigate the inhibitory effect of M1 macrophages on hair growth, we performed parallel experiments in young mice. By Day 7, both M0‐ and M2‐grafted skins had entered anagen, whereas M1‐grafted skin remained in telogen, showing a marked suppression of hair follicle entry into anagen compared with controls (Figure 8D,E). GFP‐labeled, ex vivo–polarized BMDMs were transplanted onto the dorsal skin of aged mice. The transplanted skin was harvested 1 week later and stained for CD86 and CD206. Macrophages pre‐conditioned toward M1 or M2 states largely retained their respective activation signatures, exhibiting predominant CD86 or CD206 expression. In contrast, transferred M0‐like macrophages predominantly exhibited a CD86^+^ phenotype within the aged skin microenvironment (Figure 8F,G). Immunostaining showed reduced Ki67 expression in old mice after M1 transplantation versus M0/M2 (Figure 8H,I). Additionally, β‐catenin and TYRP1, critical for melanogenesis, were lower after M1 macrophage injection (Figure 8I–K). Drawing from these findings, along with those depicted in Figure 4 and flow cytometric data, we conclude that removal of macrophages from aged skin rescues aged HFs and that M1 macrophages constitute a relatively abundant population during aging. Based on these results, we propose that M1 macrophage—rather than M0 or M2 macrophages—are the subset that inhibits hair growth and melanogenesis during aging.

**FIGURE 8:**
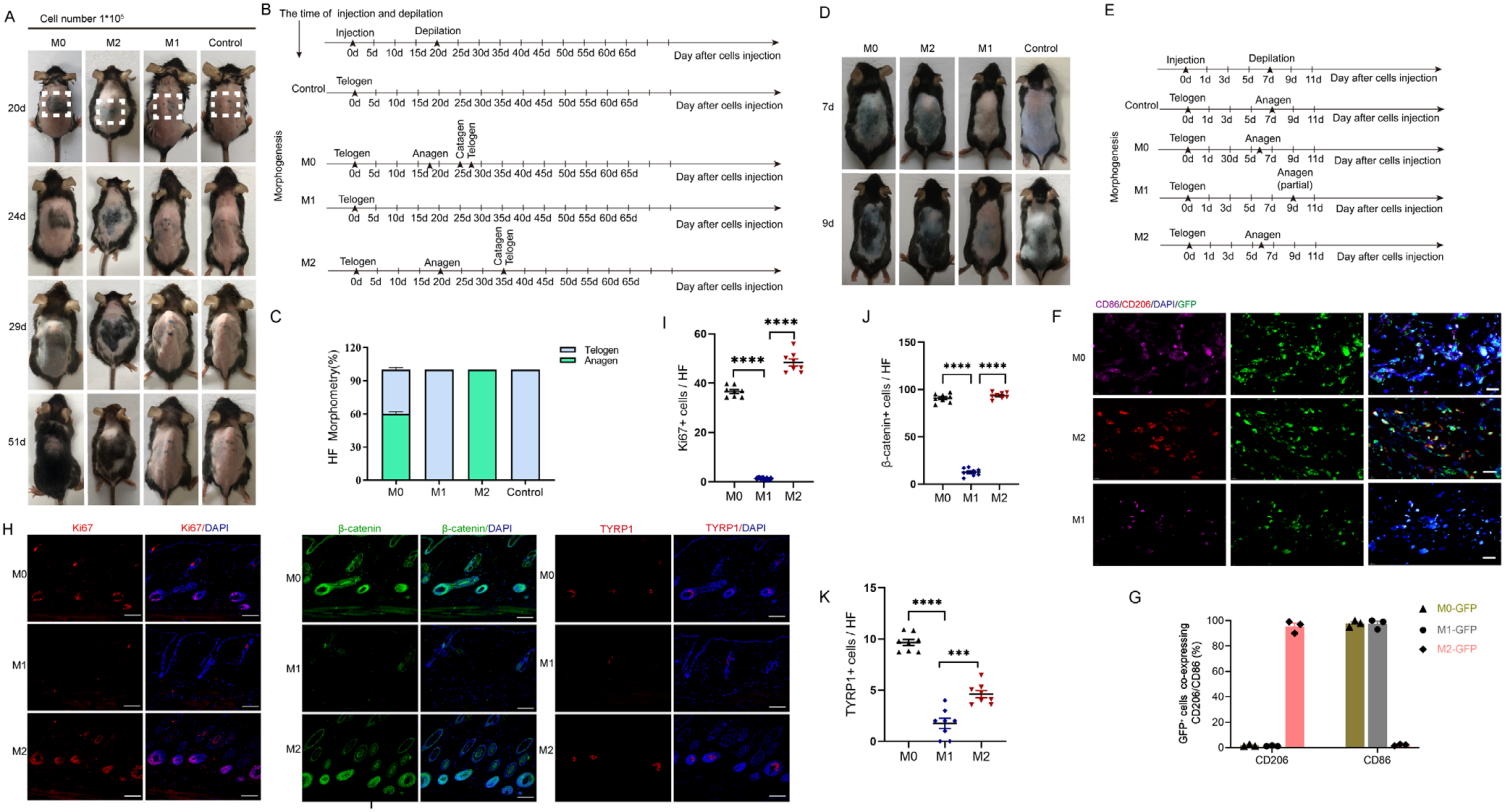
M1 macrophages inhibit the hair cycle transition. (A) Representative images of old mice (22 months) injected with different types of macrophages, *n* = 6mice per group. The white dotted area is the injection area, which is marked with invisible ink. (B) Time‐scale of the hair cycle in mice after injection with different type of macrophages. (C) Histomorphometric analysis of HF stages after injection of different types of macrophages at the Day 22, *n* = 6 mice per group. (D) Representative images of different types of macrophages injected into the whole dorsum skin of young mice at telogen state (6–7 weeks old). For the convenience of observation hair cycle, the hairs on the back skin were depilated on the Day 7 after injection. (E) Time‐scale of the hair cycle in young mice injected with different type of macrophages. (F, G) In vitro‐differentiated M0, M1 or M2 macrophages derived from GFP‐labeled bone marrow cells were transplanted into mice. After 7 days, skin sections were stained for CD86 (M1‐like) and CD206 (M2‐like). Donor GFP cells co‐localize with CD86 or CD206, enabling longitudinal tracking of macrophage subtype commitment and plasticity in vivo. The percentage of marker‐positive GFP^+^ cells in each macrophage subset was quantified, *n* = 3 biological replicates. Scale bars, 20 μm. *n* = 3. (H–K) Immunofluorescence images and quantitative analysis of Ki67, β‐catenin, and TYRP1 in skin grafts from aged mice reconstituted with M0, M1, or M2 macrophages on Day 22. *n* = 8, biological replicates; scale bars, 100 μm. ****p < 0.0001.

### 2.6 MCP‐1 Is Produced by Mature Adipocytes During Aging

Luminex cytokine panels and immunofluorescence analysis revealed increased MCP‐1 expression in dWAT within the aging mouse skin (Figure 1E, Figure 9A,B). To explore the source of MCP‐1, we performed a series of immunofluorescence co‐localization analyses. DWAT is composed of a variety of cell types. MCP‐1 could be secreted by many cell types, including preadipocytes, adipose tissue macrophages, endothelial cells, and mature adipocytes, and is also expressed during differentiation of preadipocytes into adipocytes (Gustafson et al. 2009; Starr et al. 2009; John et al. 2010; Tchkonia et al. 2010; Lumeng et al. 2011). MCP‐1 co‐localized primarily with perilipin‐positive cells (mature adipocytes) and also with F4/80‐positive cells (macrophages) and CD45‐positive cells in dWAT (Figure 9C,D). MCP‐1 expression was not detected in CD31^+^ endothelial cells or CD34^+^ cells (Figure S6). As CD34 is expressed by various cell types such as preadipocytes, fibroblasts, and adipose‐derived stem cells; therefore, these data suggest that MCP‐1 is mainly derived from mature adipocytes and macrophages during aging in dWAT. These results illustrate the potential for a vicious circle to be established, whereby MCP‐1 derived from senescent mature adipocytes increases recruitment of macrophages, which inhibits hair growth, and these macrophages further secrete MCP‐1. PPARγ is a key regulator of adipocyte differentiation and the production of mature adipocytes, and this study also found that PPARγ was upregulated in dWAT during aging (Figure 9E), which is consistent to the finding that a notable increase in dWAT thickness in aged mice reported by Salzer et al. (Sardella et al. 2018).

**FIGURE 9:**
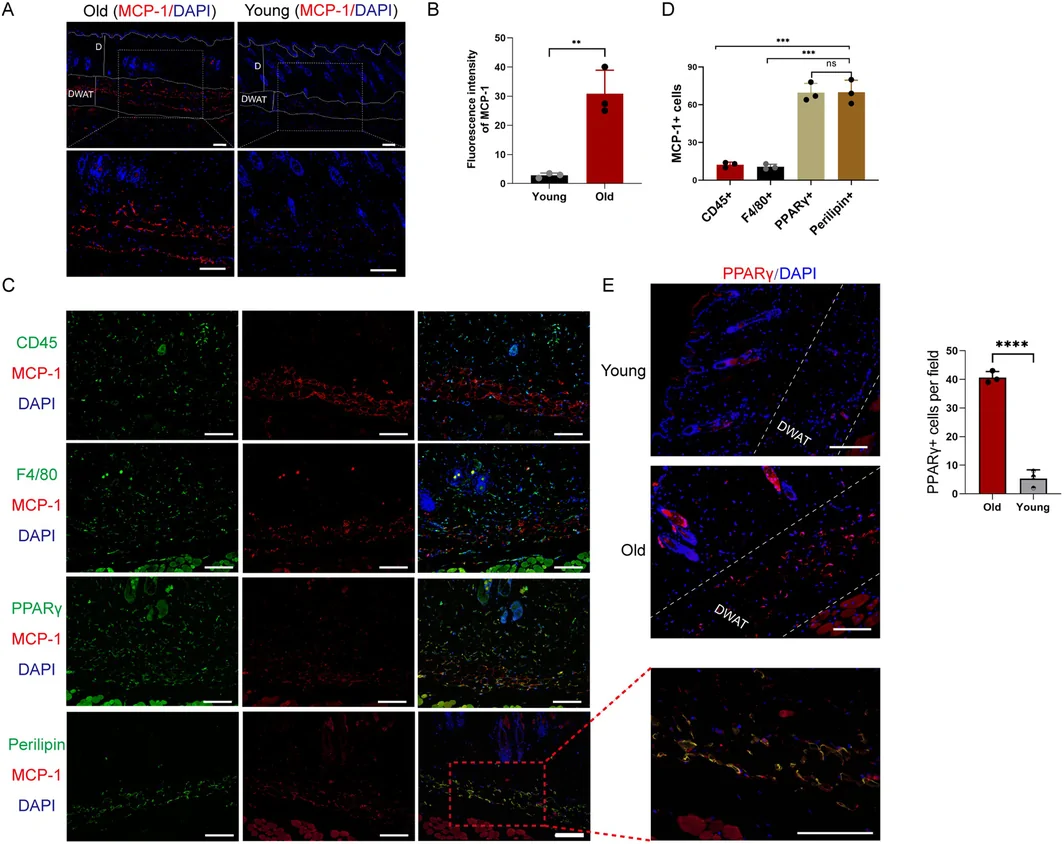
MCP‐1 is mainly derived from mature adipocytes in aging mice. (A) Representative immunofluorescence images of MCP‐1 in the skin of old and young mice, scale bar, 100 μm. (B) Quantification of MCP‐1^+^ cells in the skin of old and young groups. *N* = 3 fields per group. (C) Immunofluorescence images of co‐localization of MCP‐1 with CD45, F4/80, PPARγ, or perilipin in aging mice, scale bar, 100 μm. (D) Quantification of co‐localization of MCP‐1 with CD45^+^, F4/80^+^, PPARγ^+^, or perilipin^+^ cells in aging mice. *N* = 6 fields per group. (E) Immunofluorescence images and quantification of PPARγ (*n* = 3 fields per group) in old and young mouse skin. Scale bar, 100 μm. **p < 0.01, ***p < 0.001 and ****p < 0.0001; ns, not significant.

## 3 Discussion

Aging hair follicles are characterized by prolonged telogen, shortened anagen, and follicle miniaturization. Elucidating the mechanisms underlying these phenotypes is therefore essential for developing effective interventions. A shared hallmark of aging tissues is chronic low‐grade inflammation, largely driven by the senescence‐associated secretory phenotype (SASP), through which senescent cells release pro‐inflammatory cytokines, chemokines, and proteases (Tchkonia et al. 2010, 2013; Ghosh and Capell 2016). Given this inflammatory milieu, targeting tissue inflammation has emerged as a promising strategy to delay hair follicle aging. Aged dermal white adipose tissue (dWAT) revealed a marked upregulation of inflammatory mediators (Chen, Fan, et al. 2021; Chen, Wu, et al. 2021). In this study, we identify dWAT as a key source of MCP‐1 that couples adipose aging to HF dysfunction. Immunofluorescence co‐localization revealed that MCP‐1 is predominantly secreted by mature perifollicular adipocytes. Functionally, dWAT–derived inflammatory cues establish a chronically inflamed HF microenvironment and recruit monocytes/macrophages via the CCL2–CCR2 axis, ultimately impairing both hair cycle transition and the synthesis of key melanogenic proteins, including TYRP1, DCT, and Pmel17.

Previous studies have investigated the role of macrophages in hair growth. Macrophage numbers increased in early telogen, peaked in mid‐telogen, and then declined. Studies by Suzuki et al. indicate that macrophages may produce molecules like FGF‐5, inducing the entry of HFs into catagen (Suzuki et al. 1998). Wang et al. showed that macrophages secrete OSM to keep HFSCs in quiescence (Wang, Dai, et al. 2019; Wang, Liao, et al. 2019). Castellana et al. showed that the removal of macrophages from telogen skin promotes entry of HFs into anagen and activates β‐catenin/Wnt signaling (Castellana et al. 2014). Therefore, macrophages play a crucial role in maintaining hair telogen. Beyond the natural cycle, macrophages regulate follicle homeostasis under external stimuli and pathological conditions. Mechanical stretching and plucking induce macrophages to secrete factors that stimulate HF‐SCs. During wound healing, early infiltrating M1 macrophages mediate bacterial clearance and debris removal, whereas later‐stage M2‐polarized cells produce growth factors such as FGF2 and IGF1. These factors cooperate with WNT signaling to promote wound‐induced hair neogenesis (Chen et al. 2015; Rahmani et al. 2018; Chu et al. 2019). We observed a marked increase in macrophage abundance in aged skin, particularly within the adipose compartment. These findings are consistent with previous reports: Emilio Ortega Martinez de Victoria et al. showed that subcutaneous adipose tissue harbors more macrophages with advancing age (Ortega Martinez De Victoria et al. 2009). Carey N. Lumeng and colleagues subsequently demonstrated that aging disrupts the homeostatic balance between pro‐inflammatory and anti‐inflammatory macrophages in adipose tissue, characterized by a decline in M2‐like macrophages and upregulation of inflammatory mediators including Ccr2, Ccr5, and Il1b (Lumeng et al. 2011). The increased M1/M2 ratio indicates that aging induces both quantitative and qualitative shifts in adipose tissue macrophages (ATMs), skewing them toward a pro‐inflammatory, M1‐biased state and establishing a chronic low‐grade inflammatory environment. Notably, this phenotype closely resembles that seen in diet‐induced obesity (Lumeng et al. 2011), with our own observations. Given this context, we adopted a three‐subtype classification strategy to analyze macrophage populations. Our data show that aged skin contains more macrophages overall, with a predominance of M1‐like cells. However, accumulating evidence suggests that the classical M1/M2 binary framework is insufficient to define macrophage heterogeneity (Morris et al. 2011). Beyond canonical subsets, double‐negative (DN; CD11b^+^F4/80^+^ but lacking classic M1/M2 markers) macrophages expand significantly in aged mice (Zeyda et al. 2010). As noted above, adipose remodeling during aging parallels obesity‐associated changes (Lumeng et al. 2011), and obesity itself drives the accumulation of DN macrophages that transcriptionally activate elements of both M1 and M2 programs (Cartier et al. 2009; Zeyda et al. 2010). Importantly, similar to M1 cells, DN macrophages express high levels of CCR2 and CCR5 (Lumeng et al. 2011), which matches the chemokine receptor profile identified in our study. Our work provides evidence that physiological aging reshapes ATM biology in a comparable manner. Adoptive transfer of unpolarized macrophages stimulated hair regrowth. Donor cells subsequently acquired a CD86^+^phenotype within the skin, indicating in vivo repolarization toward an M1‐like state.

Using a mouse model, we show that DWAT–derived inflammatory cytokines establish a chronically inflamed microenvironment in the hair follicle. This triggers chemokine‐mediated macrophage recruitment, ultimately impairing hair cycle transition and the production of key melanogenic proteins (e.g., TYRP1, DCT, Pmel17), but notable anatomical differences exist between murine and human dWAT: mice bear synchronized adipose sheets divided by panniculus carnosus, whereas each human follicle forms an independent asynchronous cone‐shaped dWAT niche. Nevertheless, human ex vivo and clinical evidence confirm dWAT serves as a critical, modifiable regulator of hair growth and pigmentation. Nicu et al. (2021) demonstrated human perifollicular adipocytes secrete HGF to stimulate matrix proliferation, and atrophic dWAT surrounds miniaturized follicles in alopecia patients. Chronic perifollicular microinflammation impairs human follicles across androgenetic alopecia, alopecia areata and lichen planopilaris, with accumulated macrophages and pro‐inflammatory chemokines (Harries and Paus 2010; Harries et al. 2020). Transient mild inflammation facilitates repair, whereas senescence‐related chronic adipose inflammation suppresses hair homeostasis (Ruckert et al. 2000; Magro et al. 2011). Mice are standard preclinical models for causal signaling research, given technical limits on human scalp genetic manipulation. Though dWAT structures differ, their core follicle‐regulating pathways are comparable. Our murine observations provide mechanistic clues, which will be further verified via human HF–dWAT explants and aged scalp biopsies. Our work thus establishes an experimental foundation for understanding senescent alopecia, with the explicit recognition that ultimate clinical translation will require validation of the identified molecular targets in human scalp specimens.

## 4 Conclusion

In summary, aging orchestrates a dWAT–MCP‐1–CCR2 axis that recruits and reprograms macrophage subpopulations, disrupting the local M1/M2 balance and suppressing WNT signaling, thereby impairing both hair regeneration and production of genes associated with melanogenesis. Although the macrophage–hair follicle regulatory network remains complex, particularly regarding DN subset heterogeneity, our study offers new insight into the tripartite interplay among adipose, immune, and epithelial compartments and lays the groundwork for precision strategies targeting macrophage subsets to restore youthful hair growth and pigmentation. This study has preliminarily uncovered the mechanism by which dWAT and immune cells regulate aging‐associated hair loss from the perspective of the microenvironment and thereby highlights the microenvironment around HFs as a potential target to solve aging‐associated hair disorder.

## 5 Materials and Methods

### 5.1 Animal Experiment

Animals and treatments. Aged (18–24 months, male) and young (6–7 weeks, male) C57BL/6 mice were purchased from the Experimental Animal Center of Southern Medical University. The dorsal treatment area was in the telogen phase at baseline. Both experimental and control groups were strictly age‐matched, using mice of the same chronological age. Pioglitazone (Selleck, S2590) was dissolved in DMSO to prepare a clarified stock solution and further diluted in a vehicle containing 2% DMSO, 40% PEG300, and 5% Tween 80 in water (final concentration: 0.6 mg/mL). Mice received daily intraperitoneal (i.p.) or intradermal (i.d.) injections of pioglitazone (200 μL) or an equivalent volume of vehicle for 30 consecutive days. The PPARγ antagonist GW9662 (Selleck, S2915) was dissolved in DMSO and diluted in a vehicle composed of 5% DMSO, 40% PEG300, 5% Tween 80, and distilled water (final concentration: 200 μg/mL). GW9662 (200 μL) alone or combination pioglitazone was administered via i.p. or i.d. injection to both young and aged mice. Serum adiponectin levels were quantified using a Mouse Adiponectin (ADPN) ELISA Kit (JL20696, Jianglai Biotechnology) according to the manufacturer's instructions. Neutralizing antibodies of MCP‐1 (100 μg/10 g) were intradermally injected into the back skin of old mice in telogen phase once every 2 days for 14 days to reduce the level of MCP‐1, and every injection site was marked with invisible ink. To explore MCP‐1's inhibitory role in hair growth, we intradermally injected murine recombinant MCP‐1 (250‐10, Peprotech, 1000 ng/mL, every other day) and PBS into the entire back skin of 6–7‐week‐old mice respectively for 14 consecutive days.

### 5.2 Fluorescence‐Activated Cell Sorting

Young and aged mice were shaved and soaked in 75% alcohol for 5 min. Full thickness skin was harvested, and sWAT beneath the panniculus carnosus was removed. The remaining tissue including dWAT was then floated on 0.1% neutral protease and incubated for 60 min at 37°C. Thereafter, the epidermis was gently separated from the dermis. The harvested tissue was cut into pieces as small as possible, re‐suspended in an equivalent volume of PBS containing 0.2% collagenase, and incubated for 60 min at 37°C. The digested tissues were filtered using a 70 μm strainer. The collected cells were labeled with Fixable Viability Dye (eBioscience), an AF405‐conjugated anti‐CD45 antibody (48‐0451‐80, ThermoFisher Scientific), an APC‐conjugated anti‐CD206 antibody (17‐2061‐80, ThermoFisher Scientific), a PE‐conjugated anti‐F4/80 antibody (12‐4801‐80, ThermoFisher Scientific), and a FITC‐conjugated anti‐MHCII (11‐5321‐81, ThermoFisher Scientific) antibody for 30 min at 4°C. Flow cytometric analysis was performed using an LSRFortessa (BD Biosciences, San Jose, CA, USA) or MoFlo XDP (Beckman Coulter, Brea, CA, USA) instrument.

### 5.3 Macrophage Depletion

For macrophage depletion, old mouse skin was marked prior to treatment, and 50 μL of clodronate liposomes (Liposoma BV) or control liposomes was injected into the marked site once every 2 days.

### 5.4 Macrophage Transplantation

Primary bone BMDMs were differentiated from bone marrow isolated from 6‐ to 8‐week‐old C57BL/6 mice. Cells were cultured in RPMI 1640 growth medium containing 10% FBS, 1% penicillin–streptomycin, and 20 ng/mL recombinant murine M‐CSF (315‐02, Peprotech). Half of the medium was discarded and an equivalent volume of fresh medium was added every other day. Cells were cultured for 6 days and expressed a high level of F4/80. For M1 polarization, the medium was supplemented with 20 ng/mL mouse IFN‐γ (315‐05, Peprotech) and 100 ng/mL LPS, and cells were incubated for 24 h. For M2 polarization, 20 ng/mL IL‐4 (214‐14, Peprotech) was added to the medium and cells were incubated for 24 h. An equivalent number of polarized or unpolarized macrophages were harvested and intradermally injected into the marked sites of old mice back skin and whole young mice skin in telogen phase.

### 5.5 RNA Sequencing of the Dermis From Old Mice Treated With Clodronate Liposomes and Control Liposomes

Old mice were injected with clodronate liposomes or control liposomes. Skin was then harvested on Day 5, when both the experimental and control groups were in the telogen (resting) phase. Total RNA was extracted using the mirVana miRNA isolation kit (Ambion) according to the manufacturer's protocol. RNA integrity was assessed using the Agilent 2100 biological analyzer (Agilent Technology, Santa Clara, California, USA). According to the manufacturer's instructions, the library was constructed using the TruSeq Stranded mRNA LTSample Prep Kit (Illumina, San Diego, CA, USA). Then, sequence these libraries on the Illumina sequencing platform (Illumina Nova Seq 6000) and generate 125 bp/150 bp paired‐end reads.

### 5.6 Luminex Cytokine Panels

dWAT was harvested from pioglitazone‐treated and control mice after treatment for 30 days and analyzed using a mouse Bio‐Plex cytokine/chemokine 23‐plex array panel. Data were analyzed using the Bio‐Plex 200 System and Bio‐Plex Manager 6.0 and 6.1 software.

### 5.7 Immunofluorescence and Histology

For immunofluorescence, dorsal skin was harvested, fixed in 4% paraformaldehyde for 24 h, embedded in paraffin, and sectioned at 7 μm. After deparaffinization and heat‐induced antigen retrieval in EDTA buffer (pH 9.0), sections were incubated overnight at 4°C with the primary antibodies listed in Supplementary Table S1. Species‐appropriate fluorescent secondary antibodies are listed in Supplementary Table S1 as well. Nuclei were counterstained with DAPI. For same‐species co‐staining of F4/80 and CCR2, a TSA‐based immunofluorescence protocol was used. Images were acquired using an Olympus BX63 fluorescence microscope or a Zeiss LSM980 confocal microscope.

### 5.8 Statistics

Statistical analyses were performed using GraphPad Prism 9.0 (GraphPad Software, San Diego, CA). Data are presented as mean ± SEM. *p* < 0.05 was considered statistically significant. Normality was assessed using the Shapiro–Wilk test. For comparisons between two groups, an unpaired, two‐tailed Student's *t*‐test was used for normally distributed data; otherwise, the Mann–Whitney *U* test was applied. For comparisons involving more than two groups, one‐way ANOVA with Tukey's post hoc test was used for parametric data, and the Kruskal–Wallis test with Dunn's post hoc test was used for non‐parametric data.

## Author Contributions

J.C. and Y.H.: design, methodology, writing, data collection, and analysis; K.Y., Y.G., F.Q. and P.S.: methodology, data collection; D.L. and X.L.: data curation and formal analysis; S.W., D.D., Z.F. and D.L.: methodology and data collection; Z.H., Q.Q. and Y.M.: project administration, funding acquisition, design, supervision, and writing – review and editing. All authors agreed with the manuscript content.

## Funding

This study was supported by the National Natural Science Foundation of China (Grant No. 81701929, 81971889, 81902013, 82103763), the Guangdong Basic and Applied Basic Research Foundation (Grant No. 2019A1515012170, 2020A1515110700, 2024A1515011392).

## Ethics Statement

All experiments involving animals were conducted according to the ethical policies and procedures approved by the ethics committee of the Nanfang Hospital, Southern Medical University (Approval no. NFYY‐2018‐1053).

## Consent

The authors have nothing to report.

## Conflicts of Interest

The authors declare no conflicts of interest.

## Supporting information


**Figure S1:** Heat map of the genes about cell cycle that significantly differed between the dermis of old mice treated with clodronate liposomes (D) and the dermis of old mice treated with control liposomes (CD). *n* = 3/per group.
**Figure S2:** Pioglitazone promotes hair follicle activation independently of its adipogenic function. (A) Schematic of intraperitoneal (i.p.) and intradermal (i.d.) treatment schedules for mice receiving pioglitazone alone, PPARγ antagonist alone, or their combination. *n* = 5 per group. (B) Proportion of mice showing visible anagen entry on the dorsal skin at Day 20 after intradermal administration. (C) Representative H&E‐stained dorsal skin sections from intradermal treatment groups. (D) Quantification of dermal white adipose tissue (dWAT) thickness across treatment groups. (E) Immunofluorescence staining for MCP‐1 in dorsal skin from intradermal treatment groups. (F) Quantitative analysis of MCP‐1 fluorescence intensity. (G) Serum adiponectin levels in the indicated treatment groups of old mice. *p < 0.05;**p < 0.001;***p < 0.001;****p < 0.0001; ns, not significant.
**Figure S3:** The dermis of old mice treated with clodronate liposomes or treated with control liposomes were harvested. Their RNAs were extracted and used to perform RNA‐seq analysis to evaluate difference in gene expression at M‐Clo versus control group. (A) Histograms show a shortlist of the change of keratins‐associated genes. (B) Histograms show that the genes were involved in HF activation and pigmentation.
**Figure S4:** The top 30 enriched GO terms in the upregulated genes.
**Figure S5:** (A) Representative images of old mice (18 months) injected with different types of macrophages, *n* = 3. The white dotted area is the injection area, and black embroidery is the injection point. (B) Proportion of mice showing visible anagen entry on the dorsal skin at Day 13 and 19 after intradermal administration. *n* = 3.
**Figure S6:** Immunofluorescence images of co‐localization of MCP‐1 with CD31 and CD34 in the aging mice, Scale bar, 100 μm.

**Table S1:** Antibodies used for immunofluorescence.

## Data Availability

The RNA‐seq data presented in this study can be available here: https://www.ncbi.nlm.nih.gov/geo/query/acc.cgi?acc=GSE215878. Other data supporting the findings of this study are available in the Supporting Information.
