# Supplementary material for: MCP‐1 From Aging Dermal Adipose Tissue Impairs Hair Follicle Cycling via Macrophage Recruitment: Implications for Age‐Related Alopecia
Source: Aging Cell. 2026 Nov 8;25(11):e70799. doi: 10.1111/acel.70799 (PMC13745311; doi:10.1111/acel.70799)
Supplement: Supplementary file 1 [file ACEL-25-e70799-s001.docx]

**Supplemental Figures**

**Figure S1** Heat map of the genes about cell cycle that significantly differed between the dermis of old mice treated with clodronate liposomes (D) and the dermis of old mice treated with control liposomes (CD). n=3/per group.

Figure S2. Pioglitazone promotes hair follicle activation independently of its adipogenic function. (A) Schematic of intraperitoneal (i.p.) and intradermal (i.d.) treatment schedules for mice receiving pioglitazone alone, PPARγ antagonist alone, or their combination. n = 5 per group. (B) Proportion of mice showing visible anagen entry on the dorsal skin at day 20 after intradermal administration. (C) Representative H&E-stained dorsal skin sections from intradermal treatment groups. (D) Quantification of dermal white adipose tissue (dWAT) thickness across treatment groups. (E) Immunofluorescence staining for MCP-1 in dorsal skin from intradermal treatment groups. (F) Quantitative analysis of MCP-1 fluorescence intensity. (G) Serum adiponectin levels in the indicated treatment groups of old mice. *P < 0.05;**P < 0.001;***P < 0.001;****P < 0.0001; ns, not significant.

**Figure S3** The dermis of old mice treated with clodronate liposomes or treated with control liposomes were harvested. Their RNAs were extracted and used to perform RNA-seq analysis to evaluate difference in gene expression at M-Clo versus control group. A Histograms show a shortlist of the change of keratins-associated genes. B Histograms show that the genes were involved in HF activation and pigmentation.

**Figure S4** The top 30 enriched GO terms in the upregulated genes.

**Figure S5** (A) Representative images of old mice (18 months) injected with different types of macrophages, n=3. The white dotted area is the injection area, which is marked with invisible ink. (B) Proportion of mice showing visible anagen entry on the dorsal skin at day 13 and 19 after intradermal administration. n=3.

**Figure S6** Immunofluorescence images of co-localization of MCP-1 with CD31 and CD34 in the aging mice, Scale bar, 100 μm.
